# Oxaliplatin versus irinotecan as first-line therapy in metastatic colorectal cancer with prior adjuvant treatment: a retrospective study on efficacy, sequential therapy, and the impact of thrombocytopenia

**DOI:** 10.3389/fonc.2025.1631022

**Published:** 2025-11-17

**Authors:** Han Shan, Shuohan Huang, Changming Zhou, Mengmeng Wang, Qiong Du

**Affiliations:** 1 Department of Pharmacy, Fudan University Shanghai Cancer Center, Shanghai, China; 2 Department of Oncology, Shanghai Medical College, Fudan University, Shanghai, China; 3 Department of Cancer Prevention, Fudan University Shanghai Cancer Center, Shanghai, China

**Keywords:** metastatic colorectal cancer, oxaliplatin, irinotecan, first-line chemotherapy, adjuvant chemotherapy

## Abstract

**Background:**

The comparative efficacy of oxaliplatin versus irinotecan as first-line therapy in mCRC patients with prior adjuvant treatment remains unclear.

**Objectives:**

To compare the efficacy of first-line oxaliplatin-based versus irinotecan-based chemotherapy in metastatic colorectal cancer (mCRC) patients with prior adjuvant treatment and explore factors influencing survival outcomes.

**Methods:**

This retrospective single-center study analyzed 227 mCRC patients (2005–2014) receiving oxaliplatin (n=106) or irinotecan (n=121) as first-line therapy. Survival outcomes, treatment sequences, and adverse events were evaluated via multivariate analysis.

**Results:**

Compared with the irinotecan group, the oxaliplatin group had a numerically longer median OS (29.9 vs. 23.0 months; HR = 0.75, *p* = 0.043) but comparable PFS (9.2 vs. 9.4 months; *p* = 0.722). Subgroup analysis confirmed consistent OS benefits with oxaliplatin regardless of prior adjuvant regimens. The chemotherapy interchange rates differed significantly (47% oxaliplatin→irinotecan vs. 28% irinotecan→oxaliplatin, *p* = 0.004), although the treatment sequence did not affect OS (30.4 vs. 32.1 months; *p* = 0.351). Thrombocytopenia during prior adjuvant therapy was more common in the irinotecan group (29% vs. 15%, *p* = 0.016), which was correlated with oxaliplatin avoidance in subsequent lines. Multivariate analysis revealed that thrombocytopenia itself was not an independent risk factor but influenced treatment selection.

**Conclusion:**

Despite the limitations of a retrospective, single-center design, first-line oxaliplatin provides superior OS in mCRC patients with prior adjuvant therapy, independent of the treatment sequence. The OS disparity stems from the differential use of chemotherapy interchange, driven by oxaliplatin-induced thrombocytopenia during adjuvant treatment, which may lead to premature regimen abandonment. Clinicians should prioritize thrombocytopenia prevention and avoid arbitrary oxaliplatin discontinuation. These findings highlight the importance of managing oxaliplatin-associated toxicity to preserve subsequent treatment options, though they require validation in prospective studies.

## 1 Introduction

Globally, colorectal cancer (CRC) has the highest incidence and fatality rates, ranking second and third, respectively (1). CRC accounts for approximately 10% of all cancer diagnoses and cancer-related deaths annually (2). The incidence of CRC worldwide is predicted to increase to 25 million new patients by the year 2035 (3). More than half of the patients who are diagnosed with CRC eventually progress to metastatic colorectal cancer (mCRC). Unfortunately, most mCRC patients have unresectable disease (4, 5).

Chemotherapy, alongside surgery and radiotherapy, has been the cornerstone of mCRC treatment for decades (5–7). 5-Fluorouracil (5-FU) was the cornerstone of chemotherapy for mCRC patients from 1962–1996 (8). The treatment landscape has evolved with the approval of irinotecan (1996), capecitabine (2001), and oxaliplatin (2002) (4). Currently, oxaliplatin- or irinotecan-based regimens combined with fluoropyrimidines are standard first-line therapies for mCRC (9, 10). Clinical trials, including GOIM, GERCOR, WJOG4407G, and TRICOLORE, have demonstrated comparable efficacy between oxaliplatin- and irinotecan-based regimens in treatment-naïve mCRC patients, including more recent studies incorporating targeted agents (11, 12). However, these studies predominantly excluded patients with prior adjuvant chemotherapy, particularly oxaliplatin-based regimens (13–20).

The comparative efficacy of oxaliplatin versus irinotecan as first-line therapy in mCRC patients with prior adjuvant treatment remains unclear. Given their distinct pharmacological profiles (21, 22), regimen selection requires careful consideration (10).To address this gap, we conducted a single-center retrospective study evaluating the efficacy of oxaliplatin- versus irinotecan-based first-line chemotherapy in mCRC patients with a history of adjuvant treatment. This study uniquely explores the impact of prior adjuvant therapy on first-line outcomes and identifies influencing factors. Despite its retrospective design, this study provides valuable insights and fills a critical knowledge gap in mCRC treatment strategies.

## 2 Patients and methods

### 2.1 Patient population

Patients were identified from the Fudan University Shanghai Cancer Center database, which included 7,038 CRC patients under continuous follow-up between 2005 and 2014. Those who received oxaliplatin-based or irinotecan-based first-line therapy were reviewed. The inclusion criteria were as follows (1): histologically confirmed unresectable mCRC (2); ECOG performance status (PS) score of 0–2 (3); prior radical surgery and adjuvant chemotherapy, with ≥6 months since the last chemotherapy cycle; and (4) complete clinical and treatment records for mCRC. The data collected included age, gender, ECOG PS, primary tumor sidedness, metastatic sites, RAS/BRAF and MMR status, comorbidities, prior adjuvant therapy, radiotherapy, neoadjuvant therapy (for rectal cancer), and subsequent treatments. The outcomes were updated through May 31, 2021, via electronic medical records and telephone follow-ups.

This study was approved by the independent Ethics Committee of Fudan University Shanghai Cancer Center. All research processes were conducted in accordance with the ethical standards of the Ethics Committee of the Institute and the 1964 Helsinki Declaration and its subsequent amendments or similar ethical standards. As this study is a retrospective study, all the information collected is anonymous and there is no expected risk to participants, informed consent can be exempted. All data analyzed in this research are available and can be provided.

### 2.2 Treatment and assessment of efficacy

The oxaliplatin-based regimen included oxaliplatin combined with fluoropyrimidines (5-FU, capecitabine, tegafur-gimeracil-oteracil, or tegafur), whereas the irinotecan-based regimen comprised irinotecan with fluoropyrimidines (5-FU, capecitabine, or tegafur-gimeracil-oteracil). Both regimens could include targeted agents such as cetuximab, bevacizumab, or recombinant human endostatin. The dosage refers to the instruction manual standard dose, with no delays exceeding one cycle permitted. Treatment continued for at least six months unless disease progression occurred. Prior oxaliplatin exposure and regimen details were verified by review of chemotherapy prescription records and clinical notes in the electronic medical record. The occurrence of grade ≥2 thrombocytopenia during adjuvant therapy was ascertained from laboratory records and clinical documentation.

Overall survival (OS) and progression-free survival (PFS) were the primary endpoints. OS was defined as the time from first-line treatment initiation to death from any cause or the last follow-up. PFS was measured from first-line treatment initiation to disease progression, death, or the last follow-up (23). The first-line treatment start time was defined as the first administration of oxaliplatin or irinotecan. Efficacy was assessed via the RECIST version 1.1 criteria.

### 2.3 Statistical analysis

Fisher’s exact test or the chi-square test was used to analyze categorical variables. Continuous variables, represented as medians, were analyzed by the Wilcoxon Mann–Whitney test. The log-rank test was used to analyze the Kaplan–Meier survival curves. The factors affecting survival were analyzed via a Cox proportional hazards model, and the corresponding results are presented as hazard ratios (HRs) and 95% confidence intervals (CIs). Differences were considered statistically significant when *p* values were < 0.05. Graphical plotting was performed via GraphPad Prism software (version 5.01; Dotmatics, Boston, Massachusetts, United States). Statistical analyses were performed via IBM SPSS Statistics software (version 21.0; IBM Corp., Armonk, New York, United States) (24).

## 3 Results

### 3.1 Patient and treatment characteristics

**Figure 1:**
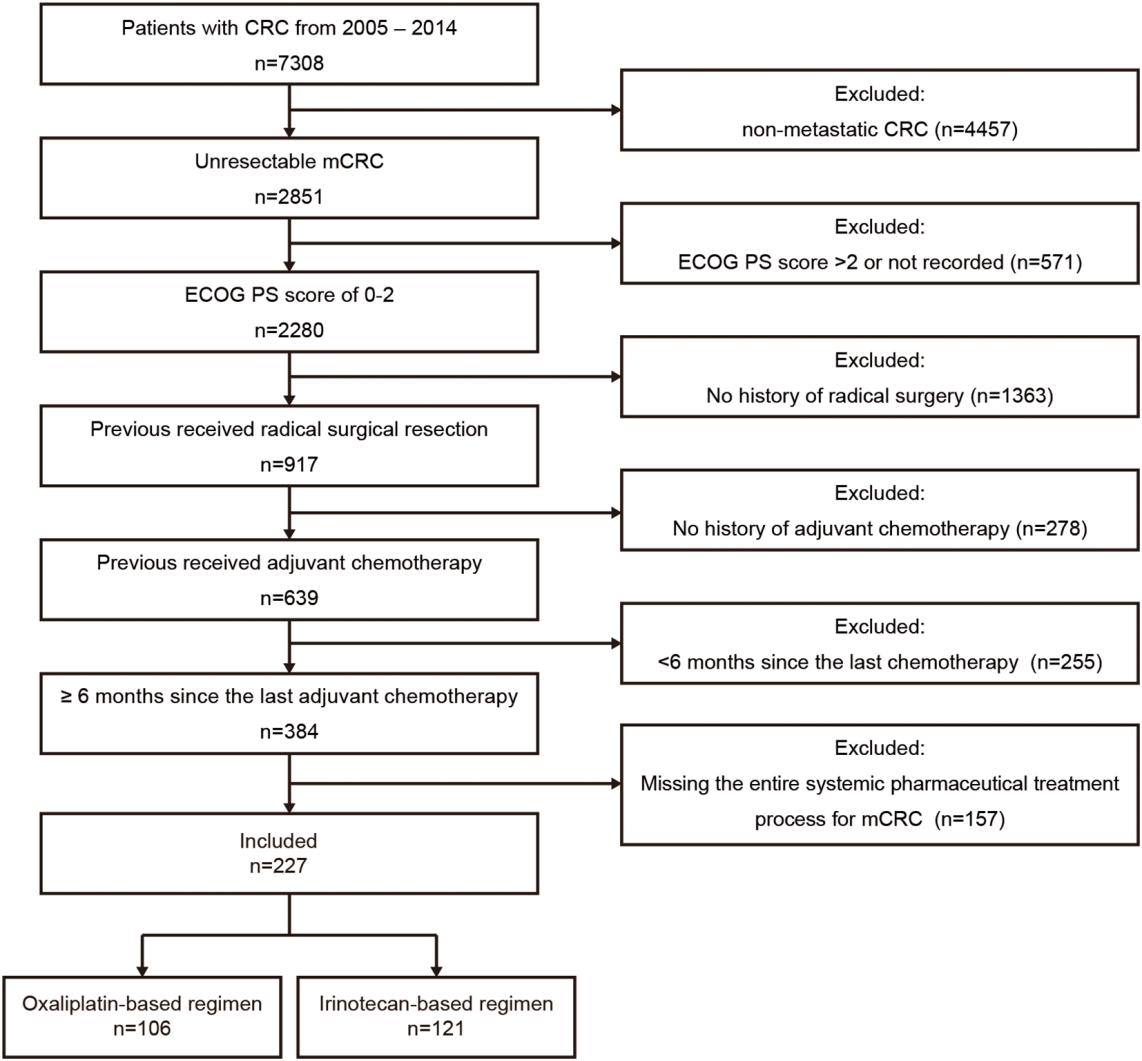
Patient selection flowchart. CRC, colorectal cancer; mCRC, metastatic colorectal cancer; ECOG PS, Eastern Cooperative Oncology Group performance status.

As illustrated in **Figure 1**, 227 Chinese mCRC patients treated with first-line oxaliplatin-based (n=106) or irinotecan-based (n=121) chemotherapy between 2005 and 2014 were selected from among 7,038 CRC patients. The median follow-up was 61.4 months (range: 3.6–169.6). Baseline characteristics, including age, gender, ECOG PS, primary tumor sidedness, metastatic sites, RAS/BRAF and MMR status, comorbidities, prior adjuvant therapy, radiotherapy, and neoadjuvant therapy for rectal cancer, are summarized in **Table 1**. No significant differences in baseline demographics were observed between the two groups. Owing to the limited availability of genetic testing in earlier years, RAS/BRAF and MMR status data were unavailable for most patients. All patients had undergone radical surgery and adjuvant chemotherapy, although details were missing for 8 patients. The use of targeted therapies in first-line treatment and additional surgery or radiotherapy for metastatic disease are detailed in **Table 2**, with no statistically significant differences observed.

**Table 1:** Characteristic of patients at baseline.

Baseline characteristics	Oxaliplatin-based n (%)	Irinotecan-based n (%)	*p* Value
Patients (n)	106	121	
Age (years)			0.244
Median	55	53	
Range	28-75	20-74	
Gender			0.671
Male	66 (62)	72 (60)	
Female	40 (38)	49 (40)	
ECOG PS			0.274
0	22 (21)	17 (14)	
1	84 (79)	103 (85)	
2	0 (0)	1 (1)	
Primary tumor sidedness			0.899
Rectum	66 (62)	72 (60)	
Left	22 (21)	26 (21)	
Right	18 (17)	23 (19)	
Metastatic sites			0.286
Lung or liver	62 (58)	62 (51)	
One site except for lung or liver	40 (38)	49 (40)	
Two or more sites	4 (4)	10 (8)	
RAS/BRAF status			0.090
RAS mutated	3 (3)	11 (9)	
BRAF mutated	1 (1)	1 (1)	
RAS and BRAF wild-type	23 (22)	13 (11)	
N/A	79 (75)	96 (79)	
MMR status			0.290
pMMR	14 (13)	23 (19)	
dMMR	14 (13)	10 (8)	
N/A	78 (74)	88 (73)	
Concomitant diseases			0.766
Hypertension	19 (18)	17 (14)	
Diabetes	13 (12)	10 (8)	
Hepatitis B	5 (5)	1 (1)	
Heart disease	3 (3)	2 (2)	
Gastritis	1 (1)	1 (1)	
Asthma	1 (1)	1 (1)	
Depression	0 (0)	1 (1)	
Previous adjuvant therapy			0.295
Oxaliplatin plus fluoropyrimidines	89 (84)	112 (93)	
Fluoropyrimidines monotherapy	6 (6)	2 (2)	
Cisplatin plus fluoropyrimidines	2 (2)	0 (0)	
Mitomycin plus fluoropyrimidines	1 (1)	0 (0)	
Irinotecan plus fluoropyrimidines	3 (3)	3 (2)	
N/A	5 (5)	3 (2)	
Previous adjuvant therapy course			0.319
Less than 3 months	0 (0)	1 (1)	
More than 3 months but less than 6 months	48 (45)	45 (37)	
Up to 6 months	58 (55)	75 (62)	
Previous radiotherapy			0.884
Yes	31 (29)	34 (28)	
No	75 (71)	87 (72)	
Previous neoadjuvant therapy for rectal cancer			0.782
Yes	6 (9)	8 (11)	
No	60 (91)	64 (89)	

ECOG PS, Eastern Cooperative Oncology Group performance status; MMR, DNA mismatch-repair; N/A, not available.

**Table 2:** The use of targeted therapy in first-line chemotherapy and surgery or radiotherapy for metastatic disease.

Treatment	Oxaliplatin-based n (%)	Irinotecan-based n (%)	*p* Value
Targeted therapy in first-line chemotherapy			0.889
Cetuximab	7 (7)	10 (8)	
Bevacizumab	7 (7)	9 (7)	
Recombinant human endostatin	3 (3)	2 (2)	
None	89 (84)	100 (83)	
Surgery			0.243
Yes	35 (33)	31 (26)	
No	71 (67)	90 (74)	
Radiotherapy			0.453
Yes	25 (24)	34 (28)	
No	81 (76)	87 (72)	

### 3.2 Efficacy

**Figure 2:**
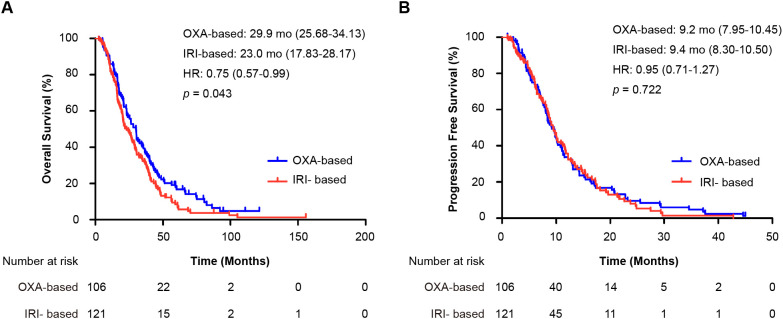
Kaplan-Meier curves for survival outcomes in the entire cohort. **(A)** Overall survival (OS) and **(B)** progression-free survival (PFS) for patients receiving first-line oxaliplatin-based (OXA) versus irinotecan-based (IRI) chemotherapy. Hazard ratios (HR) with 95% confidence intervals and *p*-values were calculated using the Cox proportional hazards model and log-rank test, respectively. OXA-based, oxaliplatin-based regimen; IRI-based: irinotecan-based regimen; HR, hazard ratio; mo: months; OS, overall survival; PFS, progression-free survival.

The OS analysis included 209 events (94 [89%] in the oxaliplatin group and 115 [95%] in the irinotecan group), whereas the PFS analysis included 187 events (91 [86%] and 96 [79%]). The median OS was associated with a borderline significant improvement in the oxaliplatin group (29.9 months, 95% CI 25.68–34.13) than in the irinotecan group (23.0 months, 95% CI 17.83–28.17; HR = 0.75, 95% CI 0.57–0.99; *p* = 0.043) (**Figure 2A**). The observed absolute difference in median OS was 6.9 months with a borderline statistical significance (*p* = 0.043), which should be interpreted with caution considering the exploratory nature of some analyses and the retrospective design. The median PFS was similar between the groups: 9.2 months (95% CI 7.95–10.45) for oxaliplatin versus 9.4 months (95% CI 8.30–10.50) for irinotecan (HR = 0.95, 95% CI 0.71–1.27; *p* = 0.722) (**Figure 2B**).

**Figure 3:**
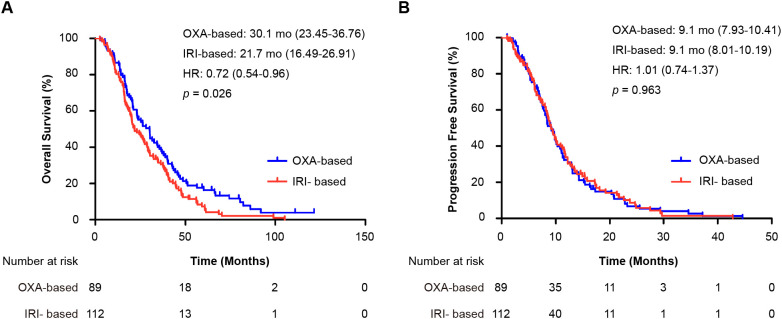
Subgroup analysis of patients who received oxaliplatin plus fluoropyrimidine as adjuvant therapy. Shown are Kaplan-Meier curves for **(A)** overall survival (OS) and **(B)** progression-free survival (PFS) comparing first-line oxaliplatin-based (OXA) and irinotecan-based (IRI) regimens. The hazard ratio (HR) and 95% confidence interval were calculated using a Cox proportional hazards model, and the *p*-value was derived from the log-rank test. OXA-based, oxaliplatin-based regimen; IRI-based: irinotecan-based regimen; HR, hazard ratio; mo: months; OS, overall survival; PFS, progression-free survival.

Subgroup analysis of patients with prior oxaliplatin plus fluoropyrimidines adjuvant chemotherapy (188 OS events, 170 PFS events) revealed consistent trends. The median OS was 30.1 months (95% CI 23.45–36.76) for oxaliplatin versus 21.7 months (95% CI 16.49–26.91) for irinotecan (HR = 0.72, 95% CI 0.54–0.96; *p* = 0.026) (**Figure 3A**). The median PFS was similar: 9.1 months (95% CI 7.93–10.41) for oxaliplatin versus 9.1 months (95% CI 8.01–10.19) for irinotecan (HR = 1.01, 95% CI 0.74–1.37; *p* = 0.963) (**Figure 3B**). These findings were consistent across all prior adjuvant therapy regimens.

### 3.3 Potential drivers for efficacy differences

To explore the superior OS outcomes with oxaliplatin-based regimens, we analyzed further-line treatments and oxaliplatin-related adverse events (**Table 3**). Patients were categorized into group A (first-line oxaliplatin) or group B (first-line irinotecan). No significant differences were observed in first-line rechallenge or subsequent targeted therapies (anti-VEGF, anti-EGFR, or anti-PD-1). However, chemotherapy interchange—switching from oxaliplatin to irinotecan or vice versa in further-line treatment—was more common in group A (47% vs. 28%, *p* = 0.004).

**Table 3:** Further-line treatments and history of oxaliplatin-related adverse events during prior adjuvant chemotherapy.

	Group A^a^ n (%)	Group B^b^ n (%)	*p* Value
Targeted therapy			0.217
anti-VEGF antibody ^c^	11(10)	18(15)	
anti-EGFR antibody ^d^	11(10)	6(5)	
anti-VEGF antibody plus anti-EGFR antibody	2(2)	6(5)	
anti-PD-1 antibody	1(1)	0(0)	
None	81(76)	91(75)	
Interchange between oxaliplatin-based and irinotecan-based regimen ^e^			0.004
Yes	50(47)	34(28)	
No	56(53)	87(72)	
First-line chemotherapy rechallenge in late-line treatment			0.268
Yes	9(8)	5(4)	
No	97(92)	116(96)	
History of oxaliplatin-induced allergy			0.424
Yes	5(5)	9(7)	
No	101(95)	112(93)	
Peripheral neurotoxicity at grade 2 or more during adjuvant chemotherapy			0.267
Yes	4(4)	9(7)	
No	102(96)	112(93)	
Thrombocytopenia during adjuvant chemotherapy			0.016
Yes	16(15)	35(29)	
No	90(85)	86(71)	

a Group A refers to patients treated with first-line oxaliplatin-based chemotherapy.

b Group B refers to patients treated with first-line irinotecan-based chemotherapy.

c Anti-VEGF antibody included bevacizumab, regorafenib, fuquitinib, apatinib, and sorafenib.

d Anti- EGFR antibody included cetuximab and panizumab.

e Chemotherapy interchange was defined as switching from a first-line oxaliplatin-based regimen to an irinotecan-based regimen in further lines, or vice versa.

f Adverse events (allergy, peripheral neurotoxicity, thrombocytopenia) during adjuvant chemotherapy were defined as grade 2 or higher according to the Common Terminology Criteria for Adverse Events (CTCAE).

**Figure 4:**
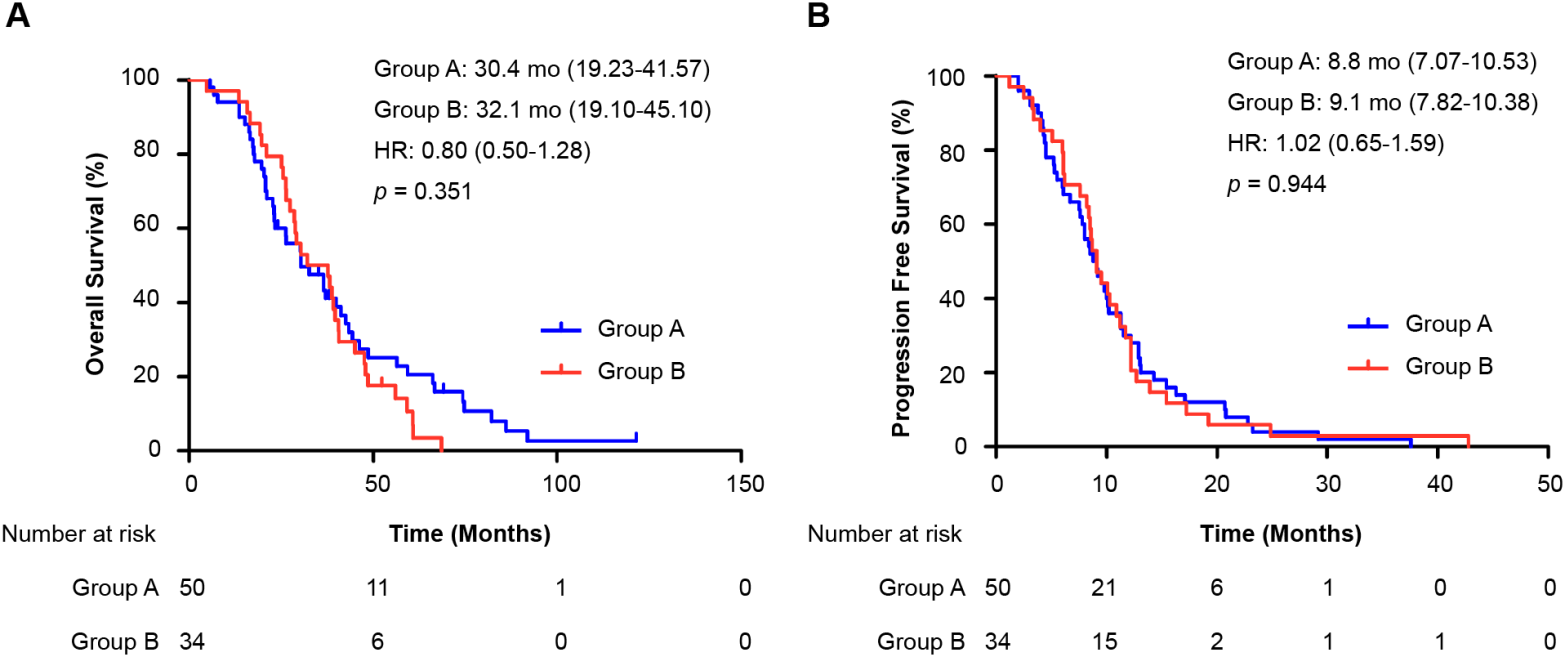
Analysis of chemotherapy interchange sequence on survival. Kaplan–Meier curves compare **(A)** overall survival (OS) and **(B)** progression-free survival (PFS) between two treatment sequences among patients who received both oxaliplatin and irinotecan. All patients had prior oxaliplatin-based adjuvant therapy. Group A: first-line oxaliplatin → further-line irinotecan. Group B: first-line irinotecan → further-line oxaliplatin. The hazard ratio (HR) was calculated from a Cox proportional hazards model.; HR, hazard ratio; mo: months; OS, overall survival; PFS, progression-free survival.

Survival analysis of patients undergoing chemotherapy interchange (78 OS events, 84 PFS events) revealed no significant differences in median OS (group A: 30.4 months, 95% CI 19.23–41.57; group B: 32.1 months, 95% CI 19.10–45.10; HR = 0.80, 95% CI 0.50–1.28; *p* = 0.351) or PFS (group A: 8.8 months, 95% CI 7.07–10.53; group B: 9.1 months, 95% CI 7.82–10.38; HR = 1.02, 95% CI 0.65–1.59; *p* = 0.944) (**Figures 4A, B**).

We also evaluated oxaliplatin-related adverse events during adjuvant chemotherapy. The rates of grade ≥2 allergies and peripheral neurotoxicity were similar between the groups, but thrombocytopenia was more common in group B (15% vs. 29%, *p* = 0.016). Multivariate analysis revealed that surgery for metastatic disease was a significant positive prognostic factor (HR = 0.71, 95% CI 0.52–0.97; *p* = 0.033), while age ≥60 years (HR = 1.37, 95% CI 1.01-1.86; *p* = 0.042) and wild-type RAS/BRAF status (HR = 0.65, 95% CI 0.43–0.99; *p* = 0.043) showed associations with OS that were of borderline statistical significance. Peripheral neurotoxicity and thrombocytopenia during adjuvant chemotherapy were not independent risk factors for OS or PFS (**Table 4**).

**Table 4:** Multivariate analyses for PFS and OS.

Variables	OS				PFS			
	Univariate		Multivariate		Univariate		Multivariate	
	HR (95% CI)	*p* Value	HR (95% CI)	*p* Value	HR (95% CI)	*p* Value	HR (95% CI)	*p* Value
Age (≧̸60 versus <60 years)	1.45 (1.07-1.96)	0.017	1.37 (1.01-1.86)	0.042	1.13 (0.81-1.58)	0.467		
Gender (Female versus male)	1.03 (0.78-1.36)	0.853			0.90 (0.67-1.21)	0.485		
Concomitant diseases (Yes versus no)	0.78 (0.58-1.05)	0.100			1.04 (0.76-1.42)	0.808		
Previous radiotherapy for primary lesion (Yes versus no)	1.22 (0.90-1.65)	0.197			1.03 (0.75-1.43)	0.851		
Previous neoadjuvant therapy (Yes versus no)	1.65 (1.01-2.68)	0.044	1.48 (0.90-2.43)	0.120	1.08 (0.65-1.82)	0.761		
Previous adjuvant therapy course (Up to 6 months versus less than 6 months)	1.07 (0.81-1.41)	0.651			1.16 (0.86-1.55)	0.335		
Two or more metastatic sites (Yes versus no)	0.92 (0.51-1.64)	0.766			1.03 (0.58-1.81)	0.931		
Surgery for metastatic disease (Yes versus no)	0.65 (0.48-0.88)	0.006	0.71 (0.52-0.97)	0.033	0.71 (0.51-0.98)	0.036	0.74 (0.53-1.02)	0.065
Radiotherapy for metastatic disease (Yes versus no)	0.95 (0.69-1.30)	0.740			0.77 (0.55-1.06)	0.112		
Peripheral neurotoxicity at grade 2 or more during adjuvant chemotherapy (Yes versus no)	0.95 (0.52-1.75)	0.874			0.84 (0.44-1.62)	0.609		
Thrombocytopenia during adjuvant chemotherapy (Yes versus no)	1.18 (0.85-1.63)	0.327			1.43 (1.00-2.06)	0.052	1.35 (0.94-1.95)	0.109
RAS/BRAF status (Wild-type versus mutated and unknown)	0.58 (0.38-0.87)	0.009	0.65 (0.43-0.99)	0.043	0.80 (0.55-1.18)	0.263		
MMR status (pMMR versus dMMR and unknown)	1.05 (0.72-1.52)	0.801			1.14 (0.77-1.70)	0.504		
Primary tumor sidedness (Rectum and left versus right)	0.84 (0.59-1.19)	0.335			0.74 (0.51-1.08)	0.117		
Targeted therapy in first-line chemotherapy (Yes versus no)	1.00 (0.69-1.45)	0.992			1.09 (0.74-1.59)	0.672		

## 4 Discussion

This study compares the efficacy of first-line oxaliplatin-based versus irinotecan-based chemotherapy in mCRC patients with prior adjuvant treatment, using retrospective data from a single center. Our findings demonstrated comparable PFS between the two regimens, aligning with prior phase III trials (13, 14, 18). However, oxaliplatin-based therapy was associated with superior OS compared with irinotecan-based therapy. Subgroup analysis of patients with prior oxaliplatin plus fluoropyrimidines adjuvant treatment mirrored these results: there was no difference in PFS, but OS was significantly longer with oxaliplatin. Notably, OS outcomes were similar between patients receiving oxaliplatin first followed by irinotecan and those receiving the reverse sequence, which is consistent with findings in treatment-naïve populations (14). The main reason for the difference in OS among the 227 patients may be the difference in further-line therapy; that is, patients in the irinotecan-based group were less likely than those in the oxaliplatin-based group were to use the interchange regimen in further-line therapy. We speculate that the reason for this phenomenon may be that some patients had experienced thrombocytopenia during adjuvant chemotherapy in the past, leading to the abandonment of oxaliplatin as a first-line therapy and subsequent treatment.

The observed dissociation between a significant OS benefit and comparable PFS is noteworthy and merits further interpretation. This phenomenon suggests that the survival advantage associated with first-line oxaliplatin is not primarily driven by a superior initial disease control mechanism, but rather by the preservation of more effective subsequent treatment options and the opportunity for longer-term therapeutic sequencing. In other words, the choice of first-line therapy indirectly influenced overall survival by shaping the entire subsequent treatment pathway. The OS disparity is due primarily to the differential utilization of chemotherapy interchange in further-line therapy. Patients in the oxaliplatin group more frequently switched to irinotecan in subsequent lines (47% vs. 28%, *p* = 0.004), whereas fewer patients in the irinotecan group adopted oxaliplatin. Survival analysis of interchange sequences revealed no significant differences in OS or PFS, reinforcing the equivalence of treatment order in these populations (14). This suggests that the survival benefit associated with first-line oxaliplatin is not driven by a superior initial disease control, but rather by more effective subsequent treatment strategies and opportunities. This is consistent with the concept that the overall treatment journey, including the availability and effective use of multiple active agents, is a stronger determinant of OS in mCRC than the choice of first-line regimen alone.

The observed difference in median overall survival, while clinically meaningful, was associated with a borderline statistical significance (*p* = 0.043). This warrants a discussion on the robustness of this finding. We consider it unlikely to be solely a chance occurrence for several reasons. First, the HR of 0.75 translates to a 25% reduction in the risk of death for the oxaliplatin-based group, an effect size that is both clinically relevant and consistent with the established efficacy of oxaliplatin in CRC. Second, this overall survival benefit was supported by a consistent and statistically stronger trend across all pre-specified subgroup analyses, particularly in the largest and most clinically relevant subgroup of patients with prior oxaliplatin-based adjuvant therapy (HR = 0.72, *p* = 0.026). The convergence of point estimates around a similar HR value across subgroups strengthens the credibility of the primary result. Therefore, while the p-value is borderline, the combination of a clinically significant HR and consistent subgroup trends suggests that the observed OS benefit represents a true effect, albeit one that should be validated in larger, prospective studies.

A critical question arises: why do irinotecan-first patients avoid oxaliplatin in further lines? While oxaliplatin-induced allergy and neurotoxicity during prior adjuvant chemotherapy showed no intergroup differences, thrombocytopenia incidence was significantly greater in the irinotecan group (15% vs. 29%, *p* = 0.016). These findings suggest that prior oxaliplatin-associated thrombocytopenia may have influenced clinicians’ or patients’ reluctance to rechallenge with oxaliplatin, potentially compromising survival. Intriguingly, multivariate analysis revealed thrombocytopenia not as an independent prognostic factor but rather as a driver of treatment selection bias, underscoring its indirect impact on outcomes through regimen avoidance. This distinction is crucial for clinical practice: the absence of an independent prognostic effect means that a history of thrombocytopenia itself does not portend a worse outcome. Instead, the subsequent decision to avoid oxaliplatin based on that history is what appears to negatively impact survival. Therefore, the clinical imperative is not to avoid oxaliplatin in patients with a prior history of thrombocytopenia, but to implement proactive management strategies to safely deliver this effective therapy in later lines, thereby overcoming the selection bias and optimizing patient outcomes.

Our findings carry direct and actionable clinical implications for the management of mCRC patients with a history of adjuvant oxaliplatin. The observed OS benefit associated with first-line oxaliplatin, contingent upon its subsequent use in later lines, underscores the critical importance of preserving this key therapeutic agent throughout the treatment continuum. The primary clinical challenge identified here is not thrombocytopenia as a direct prognostic factor, but rather its role as a significant driver of therapeutic attrition—leading to the premature and often unnecessary abandonment of oxaliplatin. Thus, our findings advocate for a treatment philosophy that prioritizes the long-term therapeutic sequence over the choice of any single first-line regimen. Ensuring patient exposure to both oxaliplatin and irinotecan through careful toxicity management appears to be a more decisive factor for optimizing survival than the initial order of their administration.

Therefore, to integrate these findings into clinical practice, we propose the following strategy: Proactive management and mitigation of oxaliplatin-induced thrombocytopenia during the adjuvant phase are paramount. This could involve (1) Implementing rigorous schedule of regular blood cell counts during and after adjuvant therapy to identify trends early (2); For patients showing a propensity for myelosuppression, considering primary prophylaxis with thrombopoietin receptor agonists in subsequent lines of therapy when rechallenging with oxaliplatin is planned (3); Emphasizing dose delays, reductions, or supportive care measures over outright regimen discontinuation at the first sign of grade 2–3 thrombocytopenia, unless clinically severe or life-threatening. By adopting a more aggressive supportive care approach, clinicians can potentially overcome the psychological and clinical hesitancy to rechallenge with oxaliplatin, thereby ensuring patients retain access to both major chemotherapeutic classes (oxaliplatin and irinotecan) and maximize their chances of receiving effective sequential therapy. This management strategy directly addresses the root cause of the observed survival disparity and is a readily implementable takeaway from our study.

A significant limitation of this study is the lack of comprehensive molecular profiling (RAS, BRAF, and MMR status) for the majority (>70%) of our patient cohort. This is largely attributable to the historical nature of the study population (treated between 2005 and 2014), a period when routine molecular testing was not yet standard clinical practice. The absence of these data introduces a substantial risk of confounding, which could challenge the internal validity of our primary finding regarding the OS benefit of first-line oxaliplatin. This is because these biomarkers are well-established prognostic and predictive factors in mCRC (25); for instance, an uneven distribution of poor-prognosis BRAF mutations or of RAS wild-type status (which predicts response to effective anti-EGFR therapy in later lines) between the groups could plausibly account for the observed survival difference. Although our available data in a small subset of patients did not show obvious imbalances (**Table 1**), the sample size was too limited to perform meaningful adjusted analyses. Consequently, our results reflect a ‘molecularly unselected’ population from an era preceding routine biomarker testing. However, given that the addition of targeted agents was balanced between the two groups (**Table 2**) and that current standard therapies still combine biological agents with these same chemotherapy backbones, we believe the overall conclusion of our study remains insightful. The observed impact of treatment sequencing and prior toxicity on therapeutic choices and survival outcomes underscores a strategic clinical dilemma that persists today, emphasizing that the proactive management of chemotherapy-related toxicities to preserve all active treatment options for sequential use is a universally relevant principle, even as the specific accompanying targeted agents continue to evolve.

Another major limitation of our study stems from its retrospective and single-center nature. This design introduces the potential for selection bias in treatment assignment, as the choice between oxaliplatin and irinotecan may have been influenced by physician preference, patient comorbidities, or unrecorded factors not captured in our analysis. The absence of granular toxicity data from the metastatic treatment lines also limits our ability to analyze how on-treatment adverse events influenced dosing, delays, and switching decisions. Although we employed multivariate analysis to adjust for available baseline characteristics, the possibility of residual confounding remains. We acknowledge that the lack of detailed data on toxicity severity and its direct impact on treatment discontinuation limits our analysis to an observational level. Furthermore, due to the retrospective nature of this study, detailed data on the precise timing of treatment switches and reasons for discontinuation (e.g., progression vs. intolerance) were not uniformly available. This limits our ability to fully assess whether differences in treatment duration or tolerability could have influenced the outcomes of the chemotherapy interchange analysis, and our conclusions in this regard should be interpreted with this caveat in mind. Therefore, our results should be confirmed by prospective, multi-center studies.

## 5 Conclusion

In conclusion, our retrospective analysis suggests that in mCRC patients with prior adjuvant therapy, initiating first-line treatment with an oxaliplatin-based regimen may be associated with a superior overall survival outcome compared to an irinotecan-based start. However, the paramount finding of this study is that this survival benefit is not driven by the inherent superiority of one agent over the other, but is critically dependent on the successful sequential administration of both oxaliplatin and irinotecan over the course of the disease. The primary driver of the observed survival disparity was the differential application of this strategy: patients starting with oxaliplatin were significantly more likely to subsequently receive irinotecan, whereas a history of oxaliplatin-induced thrombocytopenia often precluded its use in later lines for those starting with irinotecan. Therefore, the key clinical implication is that the strategic goal should be to preserve the option of using both core chemotherapies, rather than to dogmatically prioritize one first-line regimen over the other. Clinicians should focus on proactive management of toxicities, particularly thrombocytopenia, to prevent the premature loss of either oxaliplatin or irinotecan as a viable treatment option. Future treatment strategies and clinical trials in this population should be designed with this sequential, exposure-preserving approach in mind.

This study has several limitations, including its retrospective, single-center nature, which introduces potential for selection bias and unmeasured confounding, the lack of comprehensive molecular data which may impact generalizability, and the absence of detailed toxicity profiles from metastatic-line treatment.

Despite these limitations, our findings provide crucial insights for clinical practice. Clinicians should proactively manage (e.g., with close monitoring, dose modifications, or thrombopoietic agents) rather than preemptively avoid oxaliplatin in patients with a history of mild-moderate thrombocytopenia. The strategic goal should be to ensure patients receive both oxaliplatin and irinotecan sequentially whenever possible, as the order may be less important than the exposure to both. Future prospective studies in this patient population, incorporating comprehensive molecular profiling and standardized toxicity management protocols, are warranted to confirm these observations.

## Data Availability

The original contributions presented in the study are included in the article/supplementary material. Further inquiries can be directed to the corresponding authors.
